# Mucoepidermoid Carcinoma of the Minor Salivary Glands Diagnosed by High-Definition Ultrasound and Fine-Needle Aspiration: A Milan System-Based Retrospective Study

**DOI:** 10.3390/diagnostics15091182

**Published:** 2025-05-07

**Authors:** Luisa Limongelli, Marta Forte, Gianfranco Favia, Fabio Dell’Olio, Giuseppe Ingravallo, Eliano Cascardi, Eugenio Maiorano, Alfonso Manfuso, Chiara Copelli, Antonio d’Amati, Saverio Capodiferro

**Affiliations:** 1 Department of Interdisciplinary Medicine, University of Bari “Aldo Moro”, 70100 Bari, Italy; luisa.limongelli@uniba.it (L.L.); gianfranco.favia@uniba.it (G.F.); fabio.dellolio@uniba.it (F.D.); alfonso.manfuso@policlinico.ba.it (A.M.); chiara.copelli@uniba.it (C.C.); saverio.capodiferro@uniba.it (S.C.); 2 Department of Precision and Regenerative Medicine and Ionian Area (DiMePRe-J), University of Bari “Aldo Moro”, 70100 Bari, Italy; giuseppe.ingravallo@uniba.it (G.I.); eliano.cascardi@policlinico.ba.it (E.C.); eugenio.maiorano@uniba.it (E.M.)

**Keywords:** mucoepidermoid carcinoma, minor salivary glands, ultrasound, fine-needle aspiration, targeted surgery, Milan system, oral cavity

## Abstract

**Background/Objectives**: Mucoepidermoid carcinoma (MEC) is the most common malignant tumor of the minor salivary glands, often affecting the hard palate. Preoperative diagnosis and surgical planning are challenging due to anatomical complexity and limitations in sampling, generally obtained by fine-needle aspiration (FNA). This study retrospectively evaluated the diagnostic and therapeutic performance of a high-definition ultrasound (HDUS)-guided fine-needle aspiration cytology/biopsy (FNAC/FNAB) protocol in diagnosing intraoral MEC, based on the Milan System for Reporting Salivary Gland Cytopathology (MSRSGC), with the relative clinical outcomes. **Methods**: A cohort of 64 patients with histologically confirmed MEC of the minor salivary glands, treated between 2000 and 2022, was retrospectively analyzed. All patients underwent HDUS-guided FNAC/FNAB, imaging (CT, MRI, and panoramic X-ray), and subsequent surgical treatment. The cytological specimens were classified using the MSRSGC. Surgical margins, histopathological findings, lymph node status, and follow-up outcomes were recorded. **Results**: Of 64 MECs, 42 cases were finally diagnosed as low-grade (LG)/intermediate grade (IG) and 22 as high-grade (HG) carcinomas, using a two-tier histological classification system. HDUS accurately delineated the lesion size, infiltration depth, and bone proximity, with excellent correlation with surgical specimens (difference ≤ 0.6 mm). MSRSGC classification distributed the cases across all categories, with 28 classified as malignant (category VI). Repeat FNAC improved the diagnostic yield in non-diagnostic and atypical cases. FNAB confirmed the cytological findings in all cases, with immunohistochemistry investigation with Ki-67 supporting tumor grading. Surgical margins were clear in all resections. Lymph node metastases were identified in all patients who underwent neck dissection (*n* = 18), all with HG-MEC. No recurrences occurred among the LG/IG-MEC patients during a median 2-year follow-up. **Conclusions**: The combined use of HDUS and FNAC/FNAB, interpreted through the MSRSGC framework, offers a highly accurate, minimally invasive approach for preoperative diagnosis and surgical planning in intraoral MEC. HDUS-guided cytology significantly improves diagnostic reliability, particularly in LG/IG and cystic variants, facilitating tailored surgical management. Also, the clinical outcomes may support the possibility of using a simplified grading classification for two histopathological types.

## 1. Introduction

Mucoepidermoid carcinoma (MEC) is an epithelial salivary gland neoplasm first described by Volkmann in 1895 and later defined as a “mucoepidermoid tumor” by Stewart et al. in 1945. MEC was initially categorized as “relatively favorable” and “highly unfavorable” tumors based on the clinical outcomes [1]. Subsequent studies by Eversole et al. (1972) [2,3], Evans et al. (1984) [4], and Auclair et al. (1992) [5] further refined the previous classification, distinguishing MECs into low- (LG), intermediate- (IG), and high-grade (HG) subtypes based on the proportion of different cell types. This classification remains a standard reference in the World Health Organization (WHO) 2022 classification of tumors [6], although some clinicopathological studies cited in the WHO favor the use of two histological grades only (LG/IG and HG), essentially referring to the similar clinical outcomes of LG- and IG-MEC patients [7,8,9,10].

Such histological simplification could be even more suitable for minor salivary gland MECs as they are generally diagnosed in the early stage or at least when of small dimensions in contrast with those occurring in the major salivary glands [5,11,12].

In fact, in the head and neck, approximately two-thirds of MECs arise within the parotid gland, while the remaining one-third develop in the minor (intraoral) salivary glands, predominantly affecting the palate, retromolar area, floor of the mouth, buccal mucosa, lips, tongue, and jaw. Although MEC predominantly occurs in the fifth and sixth decades of life, it is also the most common malignant salivary gland tumor in younger patients [11].

Despite the WHO 2022 classification [6] providing an accurate distinction of malignant salivary gland neoplasms (SGNs), the current TNM classification remains challenging to apply universally to intraoral tumors due to potential contextual infiltration/extension to both hard and soft tissues and the relatively small size of these lesions. Additionally, preoperative histological diagnosis is often challenging due to the limited sample obtained via fine-needle aspiration (FNA) [13].

To address this issue, the Milan System for Reporting Salivary Gland Cytopathology (MSRSGC) was introduced in 2018 as a standardized classification system providing six diagnostic categories with an associated risk of malignancy (ROM) [14]. Once a diagnosis of minor salivary gland MEC is established, further challenges arise in pre-surgical evaluation. While wide-margin surgical excision is the standard treatment, and recurrence is rare, preoperative imaging using computed tomography (CT) and magnetic resonance imaging (MRI) alone may be insufficient, particularly for small tumors or lesions involving the hard palate where bone invasion is a concern [15].

In the last two decades, high-definition ultrasound (HDUS) has proven to be increasingly useful in the preoperative assessment of proliferative lesions in the oral cavity since it provides detailed information regarding lesion dimensions, margins, depth of infiltration, and proximity to vascular and other critical structures. Additionally, HDUS findings may enhance the accuracy of FNA by improving the sample quality and quantity [16].

Recent clinicopathological studies have suggested that a binary histological classification (low/intermediate-grade vs. high-grade tumors) might provide comparable prognostic stratification to the traditional three-tier system. Such simplification could improve clinical decision making, especially in early-stage intraoral tumors, where treatment-related morbidity should be minimized.

This retrospective study evaluated the diagnostic and therapeutic efficacy of our HDUS-guided FNAC/FNAB protocol in 64 cases of intraoral MEC. We assess the correlation between our findings and the MSRSGC categories, as well as the clinical outcomes of targeted surgical therapy.

## 2. Materials and Methods

This retrospective study was conducted according to the principles of the Declaration of Helsinki and the Independent Ethical Committee of the University of Bari “Aldo Moro”, which approved the study protocol (study number: 4576; code: 1443/CE). All patients provided informed consent for diagnostic and therapeutic procedures, as well as for the potential use of biological samples for research purposes. The study cohort comprised patients diagnosed with MEC of the minor intraoral salivary glands, treated with curative intent at the Complex Operating Units of Odontostomatology/Maxillofacial Surgery of the University of Bari “Aldo Moro”, between 2000 and 2022.

Patients were selected according to the following inclusion criteria: primary MEC of the minor salivary glands; preoperative FNA cytology (FNAC) and biopsy (FNAB); surgery as the primary treatment modality; and a minimum follow-up of one year post-surgery.

The diagnostic and therapeutic protocol included the following:

1. **Data collection**: Patient demographics (age, sex, and risk factors).
2. **Oral clinical examination**: WHO eight-step assessment of signs and symptoms.
3. **Panoramic radiography**: Evaluation of bone and dental involvement.
4. **Intraoral HDUS**: Performed using a GE Logic 9 ultrasound device (General Electric Healthcare, Chicaco, IL, USA) with an 18 MHz linear probe (hockey stick-shaped); to enhance the image quality in the grayscale and color Doppler modes, the probe was enclosed in a latex protection filled with US gel to eliminate air bubbles.

o Three-dimensional assessment of lesion dimensions.
o Analysis of depth of infiltration, margins, and vascular pattern.

5. **Multi-slice spiral CT** (3D reconstruction):

o Assessment of lesions in proximity to bone.
o Determination of bone involvement and anatomical relationships with key structures (e.g., maxillary sinus and nasal cavity).

6. **MRI**: Soft-tissue lesion assessment.
7. **Preoperative neck ultrasound**: Evaluation of lymph node involvement.
8. **FNAC/FNAB procedure**:

Both FNAC and FNAB were systematically performed in all cases during the same diagnostic session. The aspiration cytology was conducted first, followed immediately by the aspiration biopsy, using the same anesthetic and preparation protocol. Rapid on-site evaluation (ROSE) was not systematically adopted during the study period, mainly due to logistical and organizational limitations in the clinical setting. However, multiple passes were performed in the same session if the sample quality appeared inadequate upon gross inspection, partially compensating for the absence of ROSE.

o They were performed by the infiltration of anesthesia after mucosal cleansing with a 2% chlorhexidine or iodine solution.
o A 20 mL syringe with a 22-gauge needle was used for FNAC; FNAB was performed using a Menghini-type cutting needle (18-gauge) connected to a Cameco syringe pistol, allowing for core tissue sampling while minimizing trauma.
o Aspiration was performed using a Cameco fine-needle biopsy gun (Belpro Medical, Anjou, Canada), targeting the lesion center for small tumors and the periphery for larger ones to avoid necrotic areas.
o The samples were processed for cytological (Papanicolaou and Alcian–PAS stains) and histological (H&E, PAS, and Alcian–PAS) examination; immunohistochemical staining for Ki-67 was performed to assess the malignancy grade.

9. **Cytological classification**: Based on MSRSGC to determine ROM and guide the surgical planning.
10. **Surgical strategy**:

o LG/IG-MEC: Wide excision with ≥1 cm margins, with bone curettage or superficial cortectomy (by a piezo-surgical device) if bone involvement was observable in radiograms.
o HG-MEC: Wide resection (including bone and/or dental structures) with modified or radical neck dissection if lymph node involvement was diagnosed or suspected.

11. **Specimen analysis**:

o Gross examination, photographic documentation, and measurement.
o Comparison of preoperative HDUS and CT dimensions with surgical specimens.
o Histopathological assessment (H&E, PAS, and Alcian–PAS; immunohistochemical markers: high/low-MW cytokeratins, calponin, SMA, S-100, and Ki-67).

12. **Postoperative follow-up**:

o LG/IG-MEC: Clinical and radiological monitoring every three months for the first year, and then annually for five to ten years.
o HG-MEC: Referral to oncology for staging and adjuvant therapy if required.

## 3. Results

**Figure 1:**
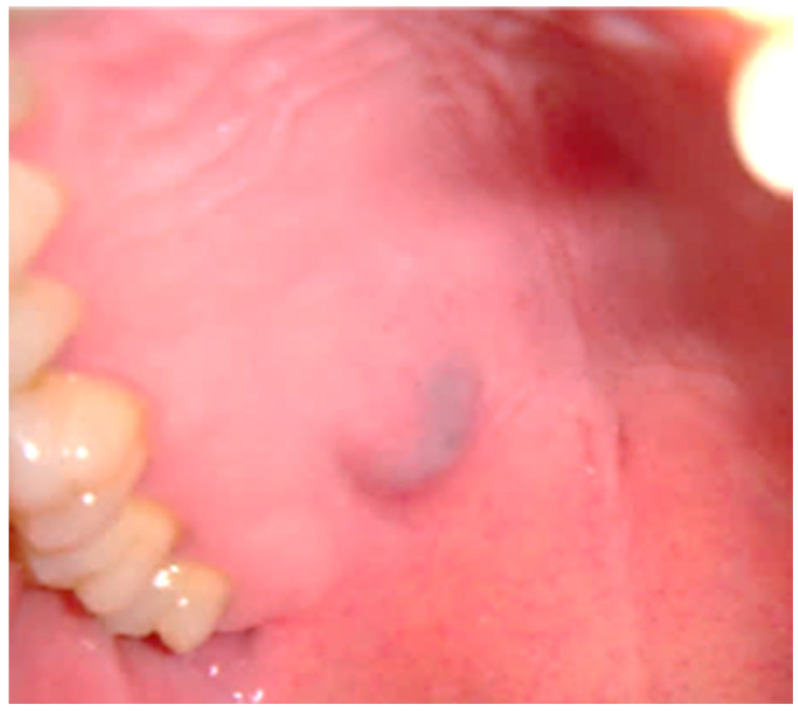
Clinical appearance of MEC of minor salivary gland of palate as firm-elastic, slow-growing, and painless swelling with bluish appearance.

**Figure 2:**
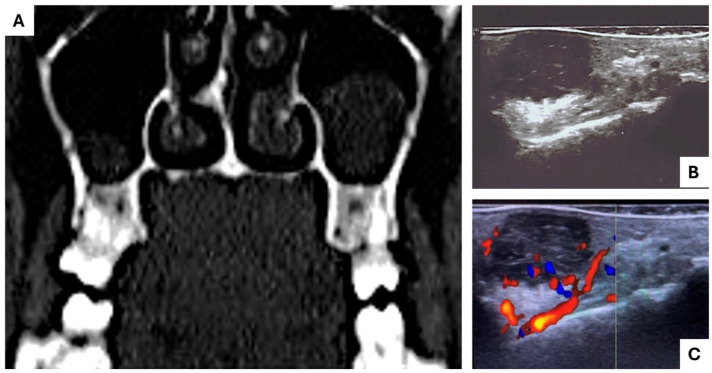
In the preoperative work-up, TC is mandatory to evaluate bone infiltration/cupping by lesions occurring in the hard palate, as shown in Figure 1 (**A**), while HDUS provides additional information regarding the lesion margin and peripheral tissue infiltration (**B**) as well the blood supply or the closeness to large vascular structures (**C**).

A total of 64 patients were included in this study, resulting in the histological examination of 42 LG/IG-MECs and 22 HG-MECs. Among the LG/IG-MECs, 29 patients were female and 13 were male, with an age of occurrence ranging from 20 to 51 years. Lesions were predominantly located in the palate (34 cases), followed by the cheek (4 cases), lips (2 cases), and tongue (2 cases). Clinically, they mainly presented as bluish nodules in 70% of cases, while the remaining 30% appeared as nodules covered by normally colored mucosa. The tumor size ranged from 1 cm to a maximum of 2.5 cm in diameter (Figure 1). HDUS consistently revealed LG/IG-MECs as encapsulated nodules with well-defined borders and a well-observable cystic space without bone resorption, also when in close proximity to the bone (e.g., the palate). CT scans confirmed the absence of bone resorption in most cases, with only a few cases exhibiting bone cupping (Figure 2). All patients with LG/IG-MECs had clinically and radiologically negative neck assessments.

For HG-MECs, 16 patients were female and 6 were male, with an age range of 31 to 68 years. The most common occurrence location was the palate (18 cases), followed by the cheek (1), tongue (1), and lips (2). Clinically, these lesions occurred as ulcers in 60% of cases and as nodules in 40%. The tumor size ranged from 1.8 cm to 4 cm. HDUS imaging typically showed HG-MECs as poorly defined nodules, often associated with bone involvement when occurring near osseous structures. HDUS features suggestive of malignancy were quantified and showed differences between tumor grades. Irregular margins were observed in 90% of HG-MECs and 10% of LG/IG-MECs. Hypoechogenicity was present in 100% of HG-MECs and 25% of LG/IG-MECs. Prominent intralesional vascularization in color Doppler was detected in 80% of HG-MECs and 15% of LG/IG-MECs. These imaging features significantly correlated with the histological grade, with high-grade tumors showing more aggressive ultrasonographic characteristics. In palatal lesions, tumor-related dental displacement was frequently observed in panoramic radiographs, while CT scans provided a detailed assessment of bone invasion and possible extension into the nasal cavity or maxillary sinus. Neck involvement was detected in 18 patients through clinical and ultrasound examination, and in 2 cases, bilateral neck dissection was performed.

All cases were preoperatively diagnosed via combined FNAC/FNAB cytology and histology. According to the MSRSGC classification, the cytological findings were categorized as follows: non-diagnostic (category I) in 6 cases, non-neoplastic (category II) in 0 cases, atypia of undetermined significance (category III) in 9 cases, benign (category IVa) in 2 cases, SGN of uncertain malignant potential (SUMP) (category IVb) in 8 cases, suspicious for malignancy (category V) in 11 cases, and malignant (category VI) in 28 cases. FNAC was repeated for all cases in categories I and III. In the two cases classified as benign (category IVa), surgery was still performed due to concerns about malignancy. In cases where FNAC was initially non-diagnostic (category I) or yielded false-positive results category IVa), HDUS findings, such as poorly defined margins, irregular vascular patterns, and hypoechogenicity, contributed to maintaining a high clinical suspicion of malignancy. This prompted repeat FNAC/FNAB or direct surgical intervention, ultimately leading to a definitive diagnosis. Such data are summarized in Table 1.

Diagnoses established by FNAC and FNAB were confirmed by final histopathological examinations in all cases. To better assess the diagnostic performance of FNAC and FNAB, we analyzed their individual and combined contribution to preoperative diagnosis. FNAC alone provided a diagnostic categorization (MSRSGC II–VI) in 85.9% (55/64) of cases in the first attempt. These included 2 cases classified as IVa, 8 as IVb, 11 as V, and 28 as VI. The remaining 9 cases—initially categorized as non-diagnostic (I) or AUS (III)—required repeat FNAC and complementary FNAB to achieve a definitive diagnosis. Table 1 has been revised to reflect both the MSRSGC distribution and the diagnostic performance of FNAC, showing the number of cases per category and the proportion successfully diagnosed in the first attempt. The combined FNAC/FNAB approach yielded a 100% preoperative diagnostic accuracy (64/64), confirming the additive value of microhistological sampling in diagnostically uncertain cases.

**Figure 3:**
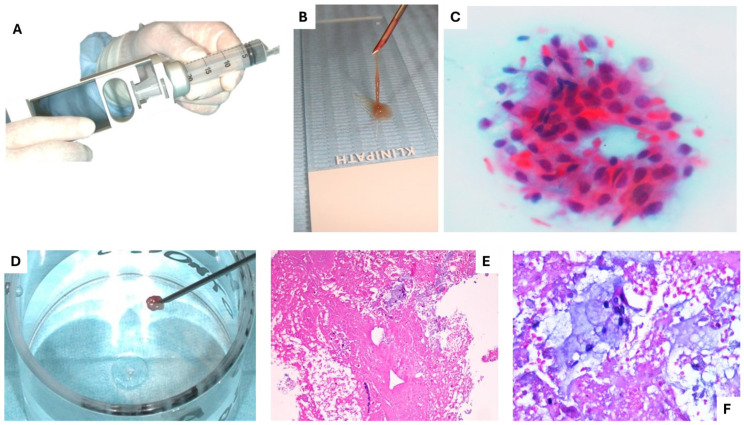
The 20 mL syringe support used for FNA (**A**). Brownish material obtained by 22-gauge needle aspiration positioned on the histological slide needs to be immediately smeared and fixed by fixation spray (**B**) to increase the readability of the cytological preparation that, when indicative of low-grade MEC, shows a cluster of epithelial cells ((**C**) Papanicolaou stain: ×20). With an 18-gauge needle, the clinician can obtain a micro-biopsy by aspiration (**D**), which may help the pathologist in the preoperative diagnosis of MEC (LG), showing, at a low power magnification, abundant amorphous eosinophilic material and few epithelial cells ((**E**) H&E stain; ×10) and showing, at a higher magnification, an intracytoplasmic mucous content ((**F**) H&E stain; ×20).

**Figure 4:**
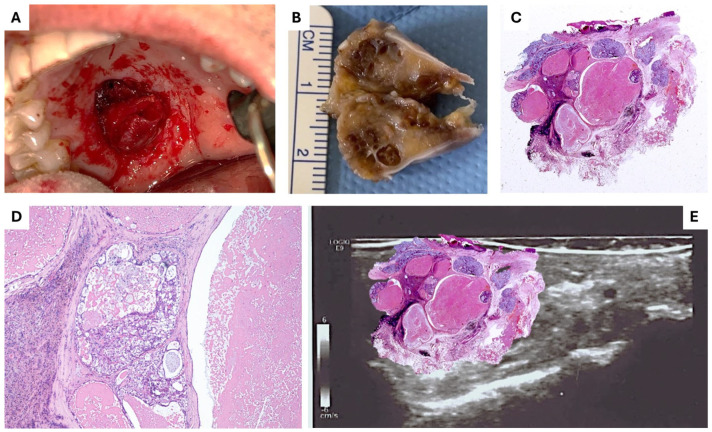
The preoperative findings led to the targeted surgical therapy (**A**); the entire tumor, the presence of cystic spaces, and the surrounding tissue are well observable if sectioned after fixation (**B**), just providing the first macroscopic information about the surgical excision. (**C**) Histologic appearance at low-power magnification of LG−MEC, showing a multi-cystic/-nodular architecture (H&E; 2×). (**D**) Closer detail, showing a cluster of malignant glands with partial mucinous secretion, located between cysts containing amorphous eosinophilic material (H&E; 100×); digital overlap of the HDUS findings and the low-power histological aspect of the tumor (**E**).

For LG/IG-MECs, FNAC revealed abundant extracellular mucoid material and small clusters of intermediate epithelial cells with regular cytological features (Figure 3). Larger mucin-producing cells contained intracytoplasmic Alcian–PAS-positive droplets. The FNAB samples confirmed these findings, highlighting large cystic mucin-filled spaces (PAS and Alcian–PAS-positive) lined by mucous and epidermoid cells with a large, pale eosinophilic cytoplasm and intermediate cells exhibiting mild cytological and nuclear atypia with minimal, mostly typical mitoses (Figure 4). Immunohistochemical analysis showed Ki-67 expression below 30%, supporting the diagnosis of LG/IG-MECs.

For HG-MECs, FNAC revealed an absence or near absence of mucoid material in the smear background, with clusters or sheets of intermediate and epidermoid cells displaying variable nuclear atypia. The FNAB samples showed a lack of cystic spaces. Histological and immunohistochemical analysis confirmed the cytological findings in all cases, allowing for ROM assessment and guiding targeted surgical management according to the MSRSGC classification.

After surgical removal, all specimens were bisected and grossly examined to visually assess the surgical accuracy. The measurements were compared with preoperative US and CT dimensional assessments, demonstrating a remarkable overlap, with differences ranging from 0.1 to 0.6 mm. Following fixation, histological examination confirmed the preoperative diagnoses in all cases, with positive staining for cytokeratins and Ki-67 (below 30% in LG/IG-MECs and 50–70% in HG-MECs) and negative staining for calponin, SMA, and S-100. The lateral and deep surgical margins were free of disease in all cases.

Lymph node metastases were identified in all 18 patients who underwent lateral neck dissection, with bilateral involvement in 2 cases. These patients received adjuvant radiotherapy, and five cases developed recurrence during follow-up. Patients with LG/IG-MECs underwent monthly follow-ups for the first year, followed by biannual assessments. No recurrences or nodal involvement were observed over a follow-up period of at least 2 years. A summary of the clinical outcomes, including survival rates and disease-free intervals, is presented in Table 2. The concordance between the HDUS measurements and the histological specimen dimensions is shown in Table 3.

## 4. Discussion

Approximately 15% of all SGNs originate in the minor salivary glands, with MEC accounting for approximately 35.9% of these cases [17]. While epidemiological studies worldwide report varying incidence rates, it is generally accepted that minor salivary gland MEC exhibits a predilection for young to middle-aged female patients, with a high occurrence in the palate, particularly the hard palate [18]. The indolent nature of MEC, characterized by slow and painless growth with a soft consistency, frequently results in delayed diagnosis. This is particularly true when tumors arise in close proximity to teeth, as they are often initially misdiagnosed as inflammatory or infectious dental or periodontal conditions [19,20].

Our findings reinforce the role of combined FNAC/FNAB guided by HDUS in achieving high diagnostic accuracy. Our findings demonstrated a 100% two-year disease-free survival in LG/IG-MECs and a strong concordance (mean dimensional difference < 0.6 mm) between preoperative HDUS and histopathological measurements. The Milan System categories strongly correlated with final histopathology, particularly after repeat FNAC in non-diagnostic and AUS cases. The systematic use of both cytological and microhistological sampling enhanced preoperative risk stratification, even in cystic and low-grade lesions where FNAC alone might be insufficient.

The current WHO (2022) definition of salivary MEC describes it as a “malignant glandular epithelial neoplasm characterized by mucous, intermediate, and epidermoid cells, with columnar, clear cell, and oncocytoid features”. Given the existence of several histopathological variants (including clear-cell, melanocytic, sclerosing, unicystic, sebaceous, psammomatous, spindle, goblet, and oncocytic types), the presence of mucin droplets, large epithelial cells with dark nuclei, and occasional inflammatory cells in FNAC/FNAB samples should raise suspicion for MEC [6].

The histopathological grading of MEC has traditionally relied on the Armed Forces Institute of Pathology (AFIP) system, which classifies tumors as LG, IG, or HG based on five key parameters: the proportion of cystic and solid components (LG-MECs show predominantly cystic areas, whereas HG-MECs are mostly solid); the presence of necrosis (absent in LG-MECs, focal in IG-MECs, and extensive in HG-MECs); perineural invasion (rare in LG-MECs and frequent in HG-MECs); nuclear atypia and anaplasia (mild in LG-MECs and pronounced in HG-MECs); and mitotic rate (low in LG-MECs and high in HG-MECs).

The 2022 WHO Classification of Head and Neck Tumors reaffirmed this grading system but emphasized the role of molecular markers in refining prognosis. The t(11;19) (q21;p13) translocation, leading to CRTC1-MAML2 fusion, is a strong prognostic indicator associated with LG/IG-MECs, which exhibit a less aggressive clinical course and better prognosis. In contrast, MAML2-negative MECs are more likely to be HG, with a higher rate of lymph node metastases and local recurrences [6,21,22].

From a cytological perspective, MEC is characterized by a mixture of mucous, epidermoid, and intermediate cells, often accompanied by abundant extracellular mucin. In FNAC, the identification of mucin droplets (Alcian–PAS-positive), large epithelial cells with hyperchromatic nuclei, and occasional inflammatory infiltrates should raise suspicion for MEC [23]. However, FNAC’s diagnostic sensitivity varies between 60% and 94%, with lower accuracy in LG-MECs, which may be misinterpreted as benign cystic lesions or reactive glandular processes [24]. FNAB provides improved diagnostic specificity over FNAC, particularly in IG and HG-MECs, as it allows for a better sampling of solid areas and necrotic components. Moreover, immunohistochemical assessment of FNAB samples for Ki-67 aids in distinguishing LG-MECs (Ki-67 < 30%) from HG-MECs (Ki-67: 50–70%), with relevant prognostic and therapeutic implications [8].

Some histological variants of MEC may pose additional diagnostic challenges: clear-cell-like MEC may resemble other clear-cell tumors such as clear-cell adenocarcinoma or metastatic renal carcinoma; sclerosing MEC may mimic reactive fibrosis, leading to underdiagnosis; and oncocytic MEC can overlap with other oncocytic SGNs, complicating differential diagnoses. Given these complexities, a combined histopathological, cytological, and molecular-grading approach is crucial for accurate diagnosis and optimal treatment planning. The MSRSGC has standardized FNAC-based categorization, providing a ROM estimation for each diagnostic category, improving predictive accuracy and reducing unnecessary surgical interventions [25,26].

In most cases, a definitive diagnosis can be achieved through FNAC/FNAB rather than requiring histological examination of the entire resected sample. FNAC has an accuracy rate of approximately 81–100% in distinguishing benign from malignant head and neck lesions, with definitive diagnoses achieved in 48–94% of cases [23,24,27]. However, FNAC can alter the tumor architecture by inducing necrosis, hemorrhage, fibrosis, and squamous metaplasia, complicating cyto-histological evaluation. Consequently, its overall diagnostic accuracy is highly dependent on the sample quality, sample quantity, and the expertise of the pathologist [28,29,30].

Beyond early clinical suspicion and a calculated ROM from the MSRSGC, the primary determinant of patient outcomes is the surgical management. Surgery should be both decisive and as conservative as possible to avoid overtreatment and related complications, particularly in young patients and those with palatal localization, which surely represents one of the most difficult areas of the oral cavity to treat. The MSRSGC provides a standardized framework for interpreting FNAC results, assigning each case to one of six diagnostic categories, each associated with a specific ROM and corresponding clinical management strategy [31]:

- Category I—non-diagnostic (ROM: 25%): Insufficient cellularity or a poor-quality sample. A repeat FNAC or core biopsy is recommended, particularly if imaging suggests malignancy.
- Category II—non-neoplastic (ROM: 10%): Includes inflammatory and reactive processes. No immediate surgery is required, but clinical follow-up is advised.
- Category III—atypia of undetermined significance (AUS) (ROM: 20%): Represents an indeterminate result requiring further evaluation. Repeating FNAC or surgical biopsy may be necessary.
- Category IVa—benign neoplasm (ROM < 5%): Typically includes pleomorphic adenomas or Warthin’s tumors, for which elective surgery may be considered.
- Category IVb—SGN of SUMP (ROM: 35%): Intermediate-risk lesions requiring histological confirmation.
- Category V—suspicious for malignancy (ROM: 60%): Surgery is recommended to confirm malignancy.
- Category VI—malignant (ROM: 90%): Confirms malignancy and necessitates definitive surgical excision.

In our study, FNAC results correlated well with final histopathology. However, categories IVb (SUMP) and V (suspicious for malignancy) required surgical resection to confirm malignancy, demonstrating the possible limitations of cytology in grading MECs preoperatively. In fact, previous studies have reported that FNAC alone may underdiagnose LG-MECs, particularly when the tumor exhibits predominantly cystic features [24]. In such cases, the use of FNAB or additional molecular markers may improve diagnostic accuracy [32]. Furthermore, while MSRSGC improves risk stratification, its predictive value varies depending on the tumor type. MECs without prominent mucous cell differentiation may be misclassified as benign or SUMP. Conversely, HG-MECs are more reliably identified, as they exhibit prominent cytological atypia [8]. Given these observations, a multimodal diagnostic approach combining cytology, molecular markers (e.g., MAML2 status), and imaging could be recommended for MEC cases in the SUMP and suspicious categories to optimize preoperative assessment and treatment planning.

As reported by Liu et al. in a comprehensive literature review, CT, MRI, and US demonstrate comparable diagnostic performance in evaluating minor salivary gland tumors [33]. Additional studies indicate that ultrasonography, particularly when combined with color Doppler imaging, offers high sensitivity and can aid in establishing a sufficiently definitive diagnosis. However, blood supply patterns alone do not significantly differentiate between benign and malignant salivary gland tumors [34]. In this study, HDUS was used alongside MRI and CT to preoperatively assess tumor dimensions, both in depth and laterally, and to evaluate infiltration margins at the time of surgical removal, especially following FNA. This approach represents a critical aspect of diagnostic and therapeutic planning for MEC patients. While CT and MRI are typically performed at the initial clinical evaluation and prior to FNA, several limitations exist, including cost, examination duration, radiation exposure (especially for young patients), and contraindications related to contrast agent use in allergic or renally impaired individuals. In contrast, ultrasonography is repeatable, well-tolerated, and minimally invasive, making it a valuable tool for evaluating or re-evaluating oral mucosal lesions (e.g., minor SGN, vascular malformations, and oral squamous-cell carcinomas) before and after FNA [35,36,37,38]. The recent literature has also highlighted the impact of ultrasound-guided FNAC in improving diagnostic reliability and reducing non-diagnostic rates in head and neck pathology [39,40].

Furthermore, high-definition imaging, particularly with specialized probes for small lesions, enables a detailed analysis of deep and lateral margins and color Doppler assessment. This provides surgeons with crucial preoperative information. Notably, in patients with a preoperative diagnosis of a LG malignant salivary tumor and no evidence of locoregional lymph node involvement, this type of ultrasonographic evaluation can guide elective, conservative surgical approaches.

The MSRSGC classification system, which has demonstrated reliability in the recent literature, was further validated by the findings of this retrospective study. Our data on survival, disease-free intervals, the strong correlation between ultrasonographic and histopathologic findings, and the nature of surgical interventions all support the efficacy of the proposed diagnostic and therapeutic protocol for minor salivary gland MECs.

This study had some limitations. First, its retrospective nature and the relatively small sample size limit the generalizability of the findings. Second, no formal statistical comparison between binary and traditional grading systems was performed. Third, the follow-up period, although sufficient to assess early recurrences, may not capture late relapses. A further limitation of our study was the lack of direct comparison between HDUS findings and CT or MRI imaging. Although HDUS proved to be highly effective in preoperative assessment, a systematic comparison with cross-sectional imaging modalities would be necessary to robustly validate its diagnostic performance. Future multicenter prospective studies with larger cohorts are warranted to validate these preliminary observations.

In conclusion, our results support the clinical reliability of a simplified two-tiered histopathological classification for intraoral MECs, as the clinical outcomes in low- and intermediate-grade tumors were largely comparable. Moreover, the strong concordance between HDUS and histological findings reinforces the value of this non-invasive imaging modality in the routine preoperative work-up. In our series, the simplified two-tiered classification demonstrated a strong clinical relevance, correlating with treatment outcomes and follow-up findings. Although a formal statistical analysis comparing two-tier versus three-tier classifications was not conducted due to the limited cohort size, the observed outcomes support the feasibility and practical usefulness of a binary approach in minor salivary gland MECs. The integration of cytological classification (via MSRSGC), high-definition ultrasound imaging, and histopathologic confirmation offers a comprehensive and accurate diagnostic approach. This strategy facilitates tailored surgical planning while minimizing overtreatment, particularly in young patients with lesions involving critical anatomical areas. Further multicenter studies with larger cohorts and molecular profiling are warranted to validate these findings and refine diagnostic protocols.

## Figures and Tables

**Table 1:** Correlation between Milan System cytology categories, final histological diagnoses, FNAC accuracy, and management in the study cohort. FNAC diagnosis in first attempt = cases where MSRSGC category II–VI was assigned without the need for repeat FNAC. The combined FNAC/FNAB approach allowed a definitive preoperative diagnosis in 100% of cases (64/64), with an FNAC alone diagnosis in 85.9% (55/64) in the first attempt.

MSRSGC Category	Cases (*n*)	Final Histological Diagnosis	FNAC Diagnosis in First Attempt (*n*)	Management
I. Non-diagnostic	6	3 LG/IG; 3 HG	0	Repeat FNAC/FNAB
II. Non-neoplastic	0	-	-	-
III. Atypia of undetermined significance (AUS)	9	7 LG/IG; 2 HG	0	Repeat FNAC/FNAB
IVa. Benign	2	2 LG	2	Surgery performed
IVb. SUMP	8	5 LG/IG; 3 HG	8	Surgery performed
V. Suspicious for malignancy	11	3 LG/IG; 8 HG	11	Surgery performed
VI. Malignant	28	24 LG/IG; 4 HG	28	Surgery performed
Total	64		55	

**Table 2:** Clinical outcomes (survival and disease-free interval).

Outcome	LG/IG-MEC (*n* = 42)	HG-MEC (*n* = 22)
Overall survival (2 years)	100%	77%
Disease-free survival (2 years)	100%	68%
Median follow-up (months)	24 (range: 12–60)	24 (range: 12–60)
Recurrences	0/42 (0%)	5/22 (22.7%)
Nodal metastasis at diagnosis	0/42 (0%)	18/22 (81.8%)

**Table 3:** Concordance between HDUS and surgical specimen dimensions. Mean difference in measurements between HDUS and histology ranged from 0.1 mm to 0.6 mm.

Parameter	Mean Lesion Diameter in HDUS (cm)	Mean Diameter in Histology (cm)
LG/IG-MEC	1.8 ± 0.5	1.7 ± 0.5
HG-MEC	2.9 ± 0.8	2.8 ± 0.8

## Data Availability

The original contributions presented in this study are included in the article. Further inquiries can be directed to the corresponding authors.

## Abbreviations

The following abbreviations were used in this manuscript:

MEC	Mucoepidermoid carcinoma
SGN	Salivary gland neoplasm
LG	Low grade
IG	Intermediate grade
HG	High grade
WHO	World Health Organization
FNA	Fine-needle aspiration
FNAB	Fine-needle aspiration biopsy
FNAC	Fine-needle aspiration cytology
MSRSGC	Milan System for Reporting Salivary Gland Cytopathology
ROM	Risk of malignancy
CT	Computed tomography
MRI	Magnetic resonance imaging
HDUS	High-definition ultrasound
H&E	Hematoxylin and eosin
AFIP	Armed Forces Institute of Pathology
SUMP	Uncertain malignant potential
